# Cardiovascular disease risk factors and cardiorespiratory fitness in persons with an intellectual disability: A seven-year observational follow-up

**DOI:** 10.17159/2078-516X/2026/v38i1a22162

**Published:** 2026-10-15

**Authors:** T Veldsman, MR de Boer, SJ Moss

**Affiliations:** 1 Physical Activity, Sport and Recreation Research focus area, Faculty of Health Sciences, North-West University, Potchefstroom, South Africa; 2 School of Health and Medical Sciences, University of Southern Queensland, Ipswich, Australia; 3 Department of Health Sciences and the EMGO Institute for Health and Care Research, Faculty of Earth and Life Sciences, VU University Amsterdam, Amsterdam, The Netherlands

**Keywords:** Intellectual disability, fitness, cardiovascular, physical activity, body composition

## Abstract

**Background:**

Compared to the general population, persons with intellectual disabilities (ID) have an increased risk of developing cardiovascular disease (CVD) and a lower life expectancy. Follow-up studies on CVD risk factors in persons with ID are lacking.

**Objectives:**

To examine long-term changes in CVD risk factors and cardiorespiratory fitness (CRF) over a seven-year period in adults with ID following participation in a prior short-term physical activity (PA) intervention.

**Methods:**

A cohort of 71 adults with ID was included in this seven-year follow-up study. Participants had the opportunity to engage in PA twice per week at a care facility. Objectively measured CVD risk factors and cardiorespiratory fitness (CRF) were assessed using a cycle ergometer physical work capacity (PWC) test.

**Results:**

After the seven-year follow-up, blood pressure (p=0.003), fat percentage (p<0.001) and cholesterol (p<0.001) values increased, while PWC decreased (p<0.001). However, the prevalence of inactivity decreased (p<0.001).

**Conclusion:**

Gains in CVD risk factors and CRF in persons with ID after a short-term PA intervention were not maintained after a seven-year follow-up. Although risk factors increased, the increase in blood pressure was not clinically relevant. Novel intervention strategies are needed to sustain improvements achieved after short-term interventions for people with ID.

Persons with intellectual disability (ID) age faster, experience poor physical and mental health and have a lower life expectancy than the general population.^[1]^ Although the life expectancy of persons with ID has increased, mortality is 20 years younger than that of the general population.^[1]^ With ageing, physical fitness and activities of daily living (ADL) decline and cardiovascular disease risk (CVD) increases.^[2]^ Persons with ID have a higher CVD risk prevalence and mortality rate than the general population, and CVD is one of the leading causes of death in the ID population.^[1,3]^ The increased CVD risk prevalence exacerbates the call for proactive prevention of CVD risk.

A quarter of the global population is physically inactive, a major health indicator and directly associated with reduced cardiorespiratory fitness (CRF) and increased CVD risk factors, such as obesity levels, which are often present in the ID population.^[4–6]^ Physical inactivity, hypertension, diabetes, obesity and dyslipidemia are common risk factors in the ID population, which could contribute to the early onset of ageing.^[3,4–8]^ People with ID have a three- to four-fold increased risk of developing diabetes and being overweight or obese, and report lower levels of CRF and lower average relative peak VO^2^ values compared to the general population.^[7,9]^ The risk of CVD could be reduced by participating in regular physical activity (PA), which improves CRF and reduces the prevalence of common risk factors associated with CVD in persons with ID.^[1]^

Exercise intervention studies investigating changes in CVD risk factors and CRF exist and range from 6 to 52 weeks; however, little data is available on the retention of the short-term changes reported.^[10,11]^ One follow-up study showed that, among persons with ID, mortality due to CVD risk is similar to that in the general population; however, the study had a small sample size and focused on CVD diagnosis rather than CVD risk and fitness.^[11]^ Given this lack of information on supervised health-based PA interventions, including follow-up data in persons with an ID, our study aimed to determine whether the short-term benefits of reducing CVD risk and improving CRF after a 12-week walking intervention in 2006 were sustained over seven years. As the present study examines long-term follow-up, the aim was not to evaluate the appropriateness of the original programme, but rather to determine whether the changes in CVD risk factors and CRF observed in 2006 were sustained seven years later. Information regarding follow-up in persons with ID remains sparse. The study could help determine whether a walking intervention leads to sustainable change in the care facility or whether alternative PA interventions are needed.

## Methods

### Study design

This study is an observational follow-up of a previously conducted 12-week walking intervention. No intervention was implemented or controlled during the follow-up period. Participants continued to receive usual care, including access to routine PA opportunities at the care facility. In 2006, all individuals (N=130) living in a care facility for persons with ID in the North West Province of South Africa were recruited. Only participants with mild to moderate levels of ID, who completed the 12-week walking intervention,^[6]^ were recruited and included in this seven-year follow-up study. All participants’ legal guardians provided consent, and the participants provided assent by writing their names on the consent form. Seventy-one participants from the 2006 study were included in this follow-up study (36 male and 35 female participants).^[6]^ Five participants passed away, 13 no longer lived in the care facility, and two participants did not complete the 2006 post-12-week intervention data collection. Participants who provided assent to the CVD risk factors but did not wish to complete the CRF test were included only in the CVD measurements (Figure 1).

Residents of the care facility live in three-bedroom apartments with a full-time caregiver. The participants were aged 25–76 years and had an intellectual age ranging from 4 to 12 years across all domains. Before participants are admitted to the care facility, a medical practitioner determines the level of ID and the participant’s intellectual age; this information was retrieved from the participant’s facility records. Participants with a physical disability, as guided by the participant’s medical record and the supervising Biokineticist, were excluded from the study.

The same data collection protocols were followed in this 7-year follow-up study as in Moss’s (2009) study.^[6]^ Details of the original 12-week walking programme implemented in 2006, including session structure, intensity, progression, and monitoring procedures, have been published previously.^[6]^ The post-intervention measurements from the 12-week walking intervention (2006) are compared to the seven-year follow-up data (2013). Familiarisation with the assessments occurred through explanations and demonstrations of the testing protocol. The Health Research Ethics Committee (NWU-00105-13-A1) reviewed and approved the study. Data collection complied with the Declaration of Helsinki.

### Cardiovascular risk factors

#### Age and smoking

Participants’ ages were collected from their medical files. Age was classified as ≥45 years in males and ≥55 years in females.^[9]^ Smoking was collected via a short yes/no question posed to the caregiver, biokineticist and the participant, and, if possible, validated in the participant’s medical file. The participants’ smoking risk was indicated as smoking or smoking within the previous six months.

#### Blood pressure and heart rate

A biokineticist measured resting blood pressure after participants had been seated for 5 minutes (Baumanometer^®^, W. Baum Co., Inc., USA) in the care facility gymnasium. Five minutes apart, two resting measurements were performed, and the average of the two measurements was recorded. The cut points for hypertension included antihypertensive medication and systolic blood pressure (SBP) >140 mmHg and/or diastolic blood pressure (DBP) >90 mmHg.^[9]^ Resting heart rate was measured with a stethoscope and stopwatch (Sprague Rappaport Type Stethoscope, China). Medication use was collected from participant files.

#### Body composition

Body composition was collected by a biokineticist in training, using standardised protocols from the International Society for the Advancement of Kinanthropometry (ISAK).^[12]^ Body mass was measured (Seca^®^700) to the nearest 0.1 kg, and height was measured with a stadiometer to the nearest 0.1 cm. Skinfolds were measured using a calibrated skinfold calliper (Harpenden^®^, UK) by a level 2 accredited kinanthropometrist. The seven-skinfold equation was used to calculate the fat percentage.^[13]^ Waist- and hip circumference (WC; HC) were measured with a non-extensible flexible steel tape. Waist-to-hip ratio (WHR) was calculated as WC divided by HC. Participants were classified as obese according to a body mass index (BMI) >30 kg/m^2^ or a waist circumference (WC in males >102 cm or females > 88 cm.^[9]^

#### Physical activity

The full-time caregiver reported the PA participation over the seven years in collaboration with the on-site supervising biokineticist. Attendance registers captured the frequency with which participants attended the on-site gymnasium for PA sessions. The PA sessions, part of the facility’s routine care, were supervised by a Biokineticist employed by the facility. Participants had the opportunity to attend two one-hour PA sessions per week, focusing on flexibility, muscle strengthening, and aerobic exercise. Physical inactivity was calculated as the proportion of persons who did not participate in regular PA.

#### Peripheral glucose and cholesterol

Glucose and total cholesterol (T-Chol) concentrations were determined from peripheral blood samples (Accutrend^®^; Roche Diagnostics, Germany) by biokineticists in training. Most participants declined peripheral blood sampling after the 12-week intervention (2006); therefore, baseline pre-intervention values were included. Dyslipidaemia was identified as T-Chol ≥5.18 mmol/L and/or a non-fasting value ≥6 mmol/L for a risk of diabetes.^[9]^ Participants using medication to stabilise glucose and cholesterol were classified as having the risk factor. This information was obtained from the participants’ caregivers and medical files.

### Cardiorespiratory fitness

The adapted submaximal YMCA bicycle ergometer test for CRF (Monark 828E, Sweden) was performed at 70% of participants’ age-predicted maximum heart rate, determined by the Karvonen formula.^[9]^ PWC was calculated as the maximum load in watts per body mass (PWC_170_/kg).^[9]^ Participants were familiar with the testing protocol and equipment through the regular, supervised PA sessions implemented at the care facility after the initial 12-week walking intervention. Not all participants completed the PWC test. Participants with three or more risk factors for CVD were classified as high risk for exercise testing.^[9]^ A 12-lead ECG (Schiller, Switzerland) was recorded and administered by a biokineticist during submaximal testing as a safety procedure. No adverse events were experienced during the testing.

### Statistical analyses

Descriptive statistics (means, standard deviations, frequencies, and prevalence) were computed for all variables (2006) and after 7 years (2013) using SPSS 31.0 (IBM SPSS Statistics, Armonk, NY). Normality was evaluated with the Kolmogorov-Smirnov test. Visual inspection of histograms and Q-Q plots, along with the Shapiro-Wilk test, was used to determine normality. Analyses were conducted using complete cases; therefore, the number of observations varied across outcomes due to missing data. The absolute change in the continuous variables was determined for CVD risk factors and CRF. Paired T-tests determined significant changes between 2006 and the 7-year follow-up. Significant changes in the frequencies of risk factors for CVD were assessed using the exact McNemar test. Effect sizes were classified as small effect: d=0.2; medium effect: d=0.5, and large effect: d≥0.8. Clinically relevant change was defined as a 10% change in cholesterol, a 5 mmHg change in SBP, or a 5 mmHg change in DBP.^[14]^ The level of significance was set at p≤0.05.

## Results

The descriptive characteristics of the participants before and after the 7-year follow-up (Table 1) indicate that, on average, the participants were middle-aged. On average, both males and females presented with higher BMI and WHR, indicative of being classified as overweight.

The pre-intervention (2006) test^[9]^ showed normal blood glucose and T-Chol levels. After seven years, the average increase in T-Chol concentration was 0.37 mmol/L in males and 0.48 mmol/L in females. Glucose levels were not sustained and increased by 0.12 mmol/L in male participants and 0.18 mmol/L in female participants, compared with baseline values.^[9]^ The post-seven-year follow-up data indicate that males performed better in CRF (0.27 W/kg) than females. The average CRF in the total group was not sustained during the observational follow-up, and CRF decreased by 0.46 watts/kg in males and 0.37 watts/kg in females.

Absolute changes in the values of the risk factors after the follow-up for the total group are presented in Table 2. After the seven years, the total group PWC values (n=45) decreased significantly by 0.43 W/kg (p<0.001; d=0.61; 17%). There is a statistically significant increase in T-chol (0.43 mmol/L; p<0.001; d=0.50; 10%); SBP (4.6 mmHg; p=0.033; d=0.264; 5%); DBP (5.5 mmHg; p<0.001; d=0.442; 10%) and body fat percentage (2.0%; p<0.001; d=0.466; 16%). Changes in CVD risk factors and CRF were observed over the seven-year follow-up, with increases in several risk markers and a decline in CRF; however, these changes were not clinically relevant or for cholesterol and diastolic blood pressure.

The distribution of risk factors for CVD (Figure 2) shows a high prevalence of overweight/obesity in 2006 and 2013 (37 and 41%, respectively). The exact McNemar test revealed a statistically significant increase in the proportion of people with ID age (p<0.001), T-Chol (p=0.001), and physical inactivity (p<0.001) after follow-up. Smoking and hypertension remained low in 2013.

## Discussion

This observational study aimed to investigate changes in CVD risk factors and CRF at a 7-year follow-up among persons with ID living in a care facility following an initial 12-week walking intervention.^[6]^ Although the initial intervention was implemented 12 years ago, the physiological mechanisms underlying CVD risk and CRF decline in adults with ID remain consistent. Because our analysis focuses on long-term within-participant change, the findings reflect underlying biological trajectories rather than context-specific programme effects. The observed changes over time suggest that short-term improvements in CVD risk factors and CRF may not persist without ongoing, structured support.

Regarding CVD risk, physical inactivity was significantly lower, whereas T-Chol was significantly higher at follow-up. Although there were statistically significant changes, they were not clinically relevant. In this observational follow-up, three-quarters of the persons with ID participated in PA, even though they had access to supervised PA twice per week. Participants had a higher SBP, DBP, body fat percentage, and cholesterol at this follow-up, and participants’ CRF generally declined after the seven-year follow-up; however, these changes were not clinically relevant. Therefore, the care facility’s usual care, short-term interventions, and increased participation in PA, including access to PA, might have helped curb the natural progression of additional lifestyle-related risk factors.

After the 7-year follow-up, a quarter of the participants were inactive, even though access to supervised PA was freely available, possibly due to a lack of interest and motivation to exercise.^[15]^ Although supervised PA sessions were available to participants twice per week as part of the facility’s usual care structure, continued access does not guarantee that individuals will change their behaviour.^[15,16]^ Thus, despite the initial intervention and the access to PA, sustained behavioural change may not have occurred. Sustained improvement depends not only on access but also on consistent engagement, sufficient intensity, and ongoing behavioural support—areas where adults with ID often face challenges. ^[16,17]^ The body of evidence related to PA interventions and persons with ID remains limited and usually focused on PA in isolation, which is insufficient to maintain long-term metabolic improvements from short-term PA interventions.^[17]^

The increase in risk factors and decrease in CRF are perhaps due to increased age, as the participants had migrated from late adulthood (38 ± 10 years) to middle age (45 to 65 years). Age and lack of PA could contribute to a decline in fitness; however, these changes are not clinically meaningful. Additionally, research indicates that improvements from short-term interventions tend to diminish unless accompanied by broader lifestyle changes (such as dietary modification, caregiver reinforcement, or more frequent activity).^[17]^ Our findings are therefore consistent with the wider literature, which shows that long-term maintenance of CVD risk reductions and CRF improvements in adults with ID requires sustained, multi-component support rather than access to supervised PA alone.^[17]^ Female participants had lower functional capacity as measured by the PWC test than male participants, possibly due to cardiovascular efficiency related to leg muscle strength.^[18]^ The decline in fitness was observed even as the prevalence of physical inactivity decreased, possibly due to ageing; however, these changes were less than the expected age-related decline, suggesting that the availability of supervised PA as part of the facility’s standard care may have helped curb it. Although there is access to supervised PA, previous literature has indicated that participants with ID have low levels of physical fitness, low levels of motivation for PA and therefore do not participate in regular PA.^[2,3,10]^

Even though physical inactivity decreased, the prevalence of overweight and obesity, T-Chol, and glucose remained high in this population; however, access to supervised PA at the facility contributed to a limited increase in risk factors, as expected with ageing. This confirms findings from previous studies reporting a high prevalence of these risk factors among persons with ID.^[1,2,5,7,8,10]^ A three-year follow-up in persons with ID has shown that physical functioning decreased by more than 50%,^[2]^ unlike our group which had less than 0.4 absolute change (nearly 20%) in their PWC after seven years, indicating that although the participants aged, their level of physical functioning only decreased about 20% after seven-years compared to the an expected 50% after 3 years from a previous study, which could indicate healthy physical ageing by the increase in PA, and the support structure implemented by the care facility.^[19]^

T-Chol increased statistically significantly among participants; however, this change was equal to the minimum clinically relevant difference. Although glucose and cholesterol concentrations were not fasting values, 45% of participants had T-Chol concentrations above 5.18 mmol/L. Females with ID are frequently prescribed contraceptive medication to prevent pregnancy and manage problems with menstruation or behaviour, which have been indicated to result in lipid changes.^[20]^ This may potentially contribute to the elevated T-Chol levels observed among our study’s female participants. Changes in cholesterol values may have been influenced by participants who declined to have cholesterol measurements after the initial 12-week intervention.^[6]^ Another factor that may contribute to the high risk of high cholesterol is participants’ dietary intake. Although everyone receives a standard diet in the care facility, the dietary intake was not recorded. The seven-year follow-up and increased participant age may also contribute to higher cholesterol levels.^[21]^

The prevalence of hypertension as a risk for CVD was low in the current study. Although mean blood pressure increased, the values remained within the normal range of 120/80 mmHg,^[9]^ and 40.5% of the individuals were overweight or obese. Persons with ID generally have lower SBP and DBP values.^[7]^ The results show a slight increase in DBP among female participants, in contrast to previous studies.^[22,23]^ This may be because persons with an ID age at a faster rate than the general population.^[3,4–8]^

In line with previous research, glucose levels increased after the seven-year follow-up, as did the prevalence of increased glucose values. The benefits of regular PA include improved glucose tolerance, reduced insulin needs, and reduced resting blood pressure. Regular PA also improves insulin sensitivity, which helps control circulating glucose concentrations.^[9]^

### Strengths and limitations

A strength of this study is that it addresses the lack of information on the sustainability of changes often found with short-term PA interventions on CVD risk factors and CRF in persons with ID living in care facilities. The study’s results should be interpreted in the context of the following limitations. During the 7 years after the short-term intervention, no annual measurements were conducted, and participants’ physical limitations were not monitored, as the study aimed only to follow up on sustained changes after the initial intervention. Nutrition or dietary data were not collected, as the facility provides a standard meal to participants. The study is based on a single facility and cannot be generalised to the broader community of people with an ID.

## Conclusion

Over a seven-year period, modest increases in CVD risk factors and declines in CRF were observed, despite reduced physical inactivity. These findings highlight the challenges of sustaining health improvements over time in adults with ID in the absence of ongoing, structured intervention. CRF decreased over the 7- year period; the decline was less than 20%, whereas a 50% decrease is expected over 3 years. The continued availability of supervised PA sessions, familiar exercise routines, and the supportive care facility environment may have contributed to greater preservation of functional capacity than typically reported in ageing adults with ID. Although the risk factors increased statistically significantly, cholesterol was clinically relevant, whereas blood pressure was not. Suggesting that access to at least 2 days of supervised PA per week may limit declines in CVD risk and CRF in persons with ID. However, future studies should focus on novel interventions that include individualised, supervised PA prescription for individuals with ID, with routine reassessment and progression to address limitations not captured in the current PA program activities, to sustain the benefits of short-term interventions over the long term for persons with ID.

## Figures and Tables

**Fig. 1:**
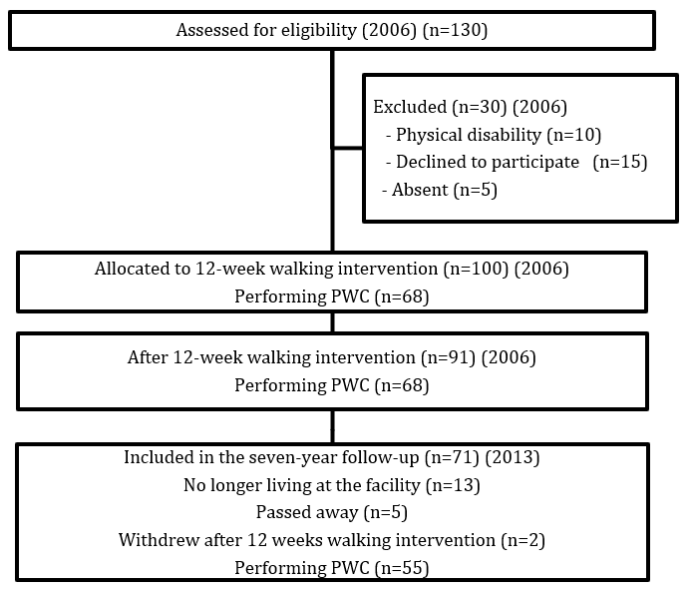
Flow diagram depicting the recruitment and inclusion of participants in the follow-up study. PWC, physical work capacity

**Fig. 2:**
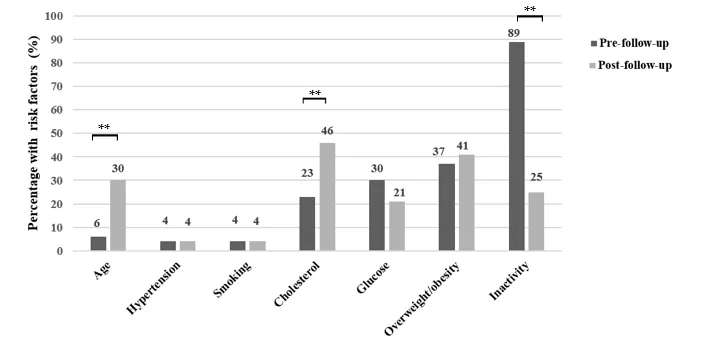
Percentage distribution of CVD risk factors before and after seven-year follow-up. **indicates significant change p<0.001.

**Table 1:** Descriptive characteristics for males and females with intellectual disability before and after a seven-year follow-up

Variables	2006 (mean±SD)		Follow-up 2016 (mean±SD)	
	Male (n=36)	Female (n=35)	Male (n=36)	Female (n=35)
**Age (years)**	37.5 ±7.4	37.3±8.4	44.7±7.3	44.4±8.5
**Height (m)**	1.71±0.11	1.59±0.10	1.70±0.11	1.59±0.09
**Body mass (kg)**	79.8±19.2	75.3±17.4	78.8±19.1	75.4±16.1
**BMI (kg/m** **^2^** **)**	27.0±5.6	29.7±6.6	27.0±5.4	29.9±6.6
**WHR**	0.88±0.07	0.79±0.10	0.88±0.06	0.80±0.06
**Body fat (%)**	17.0±6.6	23.8±6.7	20.0±6.9	24.8±5.9
**SBP (mmHg)**	110±11	112±16	116±18	115±10
**DBP (mmHg)**	71±11	69±13	73±12	75±8
**RHR (beats/min)**	82±14	84±13	80±11	81±10
**PWC (watt/kg)**	1.88±0.71	1.52±0.45	1.42±0.46	1.15±0.46
**Physical inactivity % (n)**	86 (31)	91 (32)	30 (11)	20 (7)

BMI, body mass index; DBP, diastolic blood pressure; PWC, physical work capacity; RHR, resting heart rate; SBP, systolic blood pressure; SD, standard deviations; WHR, waist-hip ratio

**Table 2:** Absolute change in cardiovascular factors after a seven-year follow-up

Variable	Absolute change	df	p-value	Cohen’s *d*	SE Cohen’s *d*	95% Confidence Intervals for Cohen’s d	
						Lower	Upper
**BMI (kg/m2)**	−0.05	69	0.86	0.02	0.05	−0.22	0.25
**Cholesterol (mmol/L)**	0.43	69	<0.001*	−0.50	0.12	−0.75	−0.25
**Glucose (mmol/L)**	0.83	69	0.76	−0.04	0.16	−0.27	0.20
**SBP (mmHg)**	4.6	67	0.03*	−0.26	0.15	−0.51	−0.02
**DBD (mmHg)**	5.6	67	<0.001*	−0.44	0.14	−0.69	−0.19
**Fat percentage (%)**	2.0	68	<0.001*	−0.47	0.08	−0.71	−0.22
**PWC (watt/kg)**	−0.43	45	<0.001*	0.61	0.19	0.29	0.92
**WHR**	0.0	69	0.71	−0.05	0.12	−0.28	0.19

* indicate significant change p≤0.05.

BMI, Body mass index; DBP, diastolic blood pressure, PWC, physical work capacity; RHR, resting heart rate; SBP, systolic blood pressure, SE, Standard error; WHR, waist-hip ratio

## Data Availability

The data supporting the findings of this study are not openly available due to institutional ethical guidelines and are available from the corresponding author, SJM, upon reasonable request.
